# Follicular regulatory T cells impair follicular T helper cells in HIV and SIV infection

**DOI:** 10.1038/ncomms9608

**Published:** 2015-10-20

**Authors:** Brodie Miles, Shannon M. Miller, Joy M. Folkvord, Abigail Kimball, Mastooreh Chamanian, Amie L. Meditz, Tessa Arends, Martin D. McCarter, David N. Levy, Eva G. Rakasz, Pamela J. Skinner, Elizabeth Connick

**Affiliations:** 1 Division of Infectious Diseases, Department of Medicine, Anschutz Medical Campus, University of Colorado, Aurora, Colorado 80045, USA; 2 Department of Surgery, School of Medicine, Anschutz Medical Campus, University of Colorado, Aurora, Colorado 80045, USA; 3 Department of Basic Science, New York University College of Dentistry, New York, New York 10010, USA; 4 Department of Pathology and Laboratory Medicine, Wisconsin National Primate Research Center, University of Wisconsin-Madison, Madison, Wisconsin 53715, USA; 5 Department of Veterinary and Biomedical Sciences, University of Minnesota, St. Paul, Minnesota 55108, USA

## Abstract

Human and simian immunodeficiency viruses (HIV and SIV) exploit follicular lymphoid regions by establishing high levels of viral replication and dysregulating humoral immunity. Follicular regulatory T cells (T_FR_) are a recently characterized subset of lymphocytes that influence the germinal centre response through interactions with follicular helper T cells (T_FH_). Here, utilizing both human and rhesus macaque models, we show the impact of HIV and SIV infection on T_FR_ number and function. We find that T_FR_ proportionately and numerically expand during infection through mechanisms involving viral entry and replication, TGF-β signalling, low apoptosis rates and the presence of regulatory dendritic cells. Further, T_FR_ exhibit elevated regulatory phenotypes and impair T_FH_ functions during HIV infection. Thus, T_FR_ contribute to inefficient germinal centre responses and inhibit HIV and SIV clearance.

## Abstract (web summary)

Antibody responses are impaired during HIV and SIV infection. Here the authors show that viral infection contributes to this process by multiple mechanisms acting on regulatory T follicular cells, including their expansion and increased suppression of the follicular T helper cells.

## Graphical abstract

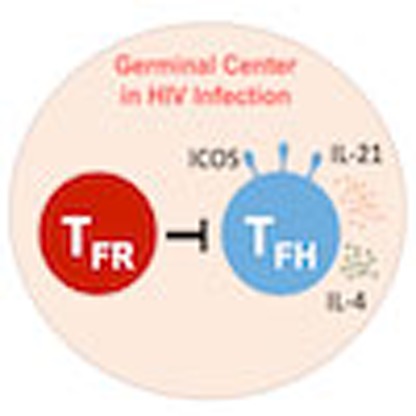

HIV rapidly establishes a productive viral infection in secondary lymphoid tissues, which is maintained during chronic stages of disease^1^,^2^. During chronic HIV infection, viral replication is highly concentrated within B-cell follicles in follicular T helper cells (T_FH_)^3^,^4^,^5^. T_FH_ are crucial initiators of the germinal centre (GC) response^6^,^7^. T_FH_ have a distinct developmental pathway characterized by Bcl-6 expression, which is dependent on inducible T-cell costimulator (ICOS) expression^8^, and produce interleukin (IL)-21 and IL-4 that together optimally drive B-cell affinity maturation and antibody specificity^9^,^10^. ICOS expression on T_FH_ is crucial for both T_FH_ differentiation and immune function^8^. An expansion of T_FH_ cells has been observed in HIV infection^11^ and simian immunodeficiency virus (SIV) infection^12^, yet this expansion does not correlate with improved GC responses. Rather, it has been shown that T_FH_ exhibit impaired activity, partly due to PD-1 ligation, manifested by reduced ICOS expression and inadequate production of IL-21 during HIV infection^13^. It remains unclear whether additional factors may drive the dysregulation of T_FH_ during HIV and SIV infection.

It has recently come to light that B-cell follicles contain a novel subset of regulatory T cell (Treg), termed follicular regulatory T cells (T_FR_)^14^,^15^,^16^. T_FR_ display a unique transcriptional pattern overlapping that of both T_FH_ and Treg, notably with combined expression of Bcl-6, Foxp3 and Blimp-1. T_FR_ originate from Treg precursors, express CXCR5 and regulate GC responses through interactions with T_FH_^14^,^15^,^16^. These studies were performed in mouse models, however, and the presence or function of T_FR_ have not yet been described in HIV or SIV infection. Some^17^,^18^,^19^,^20^,^21^, but not all^22^,^23^,^24^,^25^ studies suggest proportional, not numerical, Treg increases in the peripheral blood of HIV-infected individuals. Studies in lymph nodes (LNs) and the spleen consistently suggest proportional increases of Treg in the context of HIV or SIV infection^26^,^27^,^28^, although absolute numbers have not been determined. The impact of Treg on HIV infection is controversial with some studies suggesting that Treg exert a beneficial effect by limiting autoimmunity, HIV replication and CD4^+^ T-cell depletion^17^,^18^,^24^,^25^, whereas others suggest that Treg have a detrimental effect by inhibiting HIV-specific immune responses and causing disease progression^20^,^21^,^28^,^29^. Although it is reported that Treg from HIV-infected individuals have lower suppressive capacity than those from uninfected individuals^30^, it has also been reported that HIV binding to Tregs enhances their suppressive activity and lymphoid homing^31^. Thus, understanding the role of Treg in HIV infection is still evolving^32^, and virtually nothing is known about T_FR_ number and function in HIV infection. Here, we provide evidence for HIV-mediated T_FR_ expansion and the role of T_FR_ in T_FH_ dysregulation during HIV and SIV infection.

Through analyses of secondary lymphoid tissues from chronically HIV-infected humans and chronically SIV-infected rhesus macaques, as well as HIV infection *ex vivo* of human tonsils, we find that T_FR_ are expanded both proportionally and numerically during infection. This expansion is due to a combination of factors, including viral entry and replication, Treg acquisition of CXCR5, transforming growth factor (TGF)-β signalling, T_FR_ proliferation, low apoptosis rates and increased regulatory dendritic cell (DC) activity. In addition, we demonstrate that T_FR_ suppress T_FH_ activity during infection by inhibiting T_FH_ proliferation, IL-21 and IL-4 production and downregulating T_FH_ ICOS expression. The identification of this potent regulator of GC dynamics provides a new therapeutic target for enhancement of anti-viral humoral immunity and vaccine efficacy to promote clearance of HIV.

## Results

### T_FR_ are increased in chronic HIV and SIV Infections

**Figure 1:**
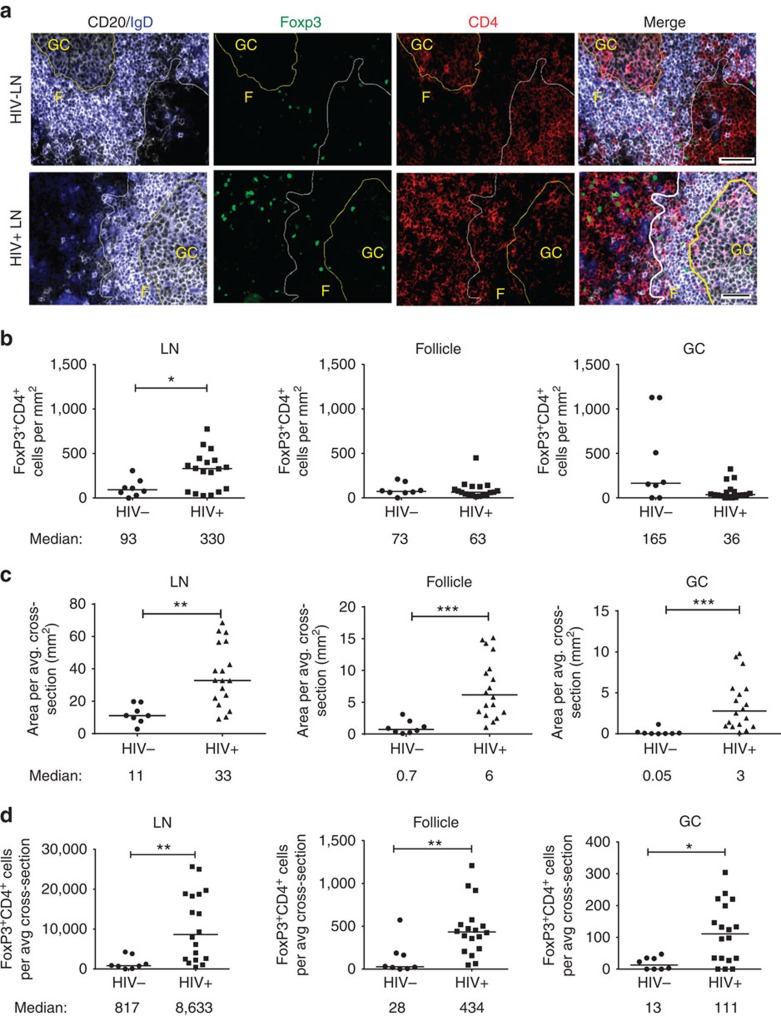
T_FR_ expansion in human lymph nodes (LNs) during HIV infection. (**a**) Representative images of immunofluorescently stained LN sections from a subject with chronic, untreated HIV infection (*n*=17) and an uninfected control subject (*n*=8). LNs were stained with fluorescently labelled antibodies to CD20 (white), IgD (blue), Foxp3 (green) and CD4 (red). Follicle (F) was defined as CD20^+^ (white line) and germinal centre (GC) was defined as CD20^+^IgD^−^ (yellow line). Images were scanned at × 60 magnification and scale bars equal 20 μm. (**b**) Foxp3^+^CD4^+^ cells were quantified in different regions of immunofluorescently stained LN shown in **a** from uninfected (*n*=8) and HIV-infected (*n*=17) subjects using visual inspection and quantitative image analysis to determine areas. (**c**) The average areas of total (LN), follicular and GC regions per LN cross-section were determined by quantitative image analysis. (**d**) The average number of CD4^+^Foxp3^+^ cells per LN, F and GC cross-section was calculated by multiplying the frequency of CD4^+^Foxp3^+^ cells per mm^2^ (**b**) by the average area of each region (**c**) for each subject. The horizontal bars of each graph indicate the median value and are listed where appropriate for clarity. Statistical analyses were performed by Mann–Whitney (Wilcoxon) tests to compare unpaired, nonparametric values and significance is denoted by asterisks where *P<0.05 and **P<0.01.

To determine if T_FR_ were present in human lymphoid tissues, we immunofluorescently labelled LN tissue cross-sections from HIV uninfected and HIV-infected individuals with antibodies to CD4, Foxp3, CD20 and IgD. CD4^+^Foxp3^+^ cells were readily detected throughout the LNs including follicular and GC regions, as shown in representative images (Fig. 1a and Supplementary Fig. 1a). Next, we quantified the number of CD4^+^Foxp3^+^ cells in total LN, follicular (CD20^+^) and GC (CD20^+^IgD^−^) regions using two to three tissue cross-sections per subject. In total LN, the frequencies of CD4^+^Foxp3^+^ cells per mm^2^ were significantly higher in HIV-infected individuals compared with uninfected controls, but frequencies of CD4^+^Foxp3^+^ in follicular and GC regions did not differ significantly (Fig. 1b). LN enlargement is a hallmark of HIV infection, and therefore frequencies of CD4^+^Foxp3^+^ cells per mm^2^ underestimate the true differences in absolute numbers of CD4^+^Foxp3^+^ cells. The average LN, follicular and GC areas in LN cross-sections, as determined by quantitative image analysis, were significantly larger in HIV-infected individuals than seronegative individuals (Fig. 1c). We then calculated the number of CD4^+^Foxp3^+^ cells per average LN tissue cross-section as well as within each compartment. Absolute numbers of CD4^+^Foxp3^+^ cells per average cross-section were significantly increased in HIV-infected individuals compared with HIV-uninfected individuals in all regions (Fig. 1d). The percentage of tissue area that stained positively for CD4 did not differ between HIV seropositive and seronegative individuals, indicating that these individuals had not yet experienced profound CD4 depletion in the LN (Supplementary Fig. 1b). In HIV-infected subjects, the percentage of activated (HLA-DR^+^CD38^+^) LN CD4^+^ T cells, as previously determined by flow cytometry^33^, did not correlate with number of Treg or T_FR_ and was inversely correlated with GC T_FR_ in the average tissue cross-section (Supplementary Fig. 1c).

**Figure 2:**
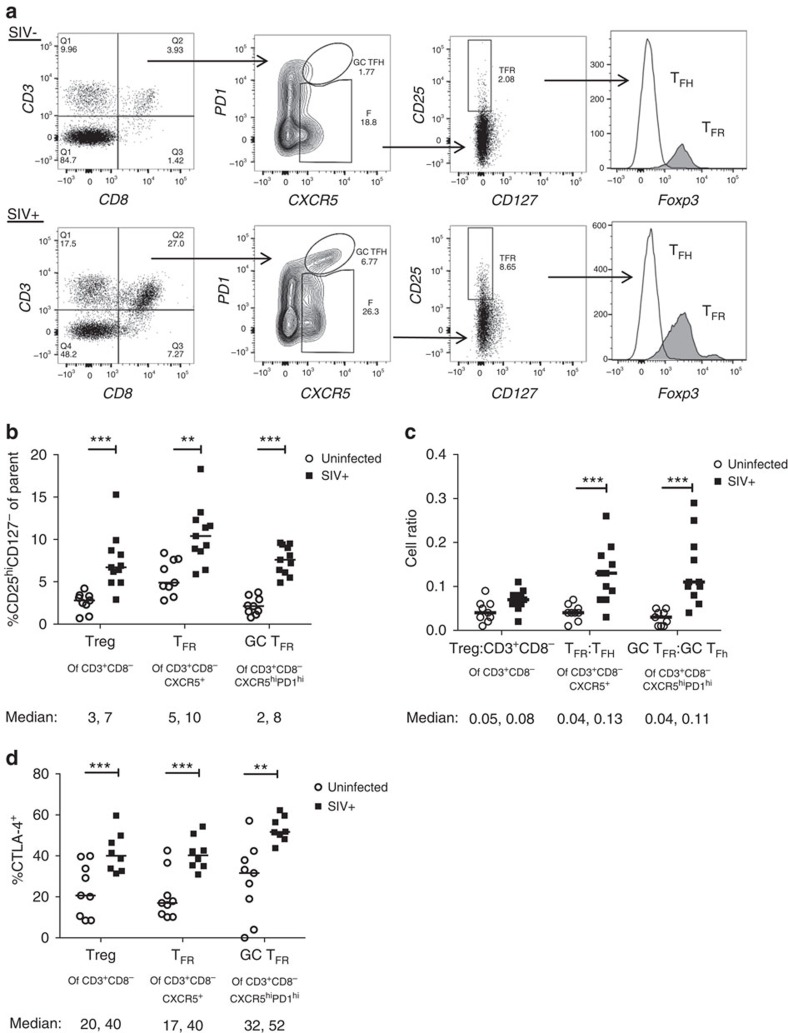
T_FR_ expansion in lymphoid tissues during chronic SIV infection. (**a**) Disaggregated lymph node and spleen cells from SIV uninfected (*n*=9) or chronically SIV-infected rhesus macaques (*n*=11) were analysed by flow cytometry. Representative examples of flow cytometry gating are shown. Of viable CD3^+^CD8^−^ cells, follicular subsets were defined as CXCR5^+^ cells (F) and germinal centre subsets were defined as CXCR5^hi^PD-1^hi^ cells (GC). Of these subsets, regulatory cells were defined as CD25^hi^CD127^−^. T_FR_ (CXCR5^+^CD25^hi^CD127^−^) were Foxp3^+^, whereas T_FH_ (CXCR5^+^CD25^lo/−^) were Foxp3^–^. (**b**) The percentages of each rhesus macaque regulatory subset, as analysed in **a** are shown. (**c**) The ratios of each regulatory cell population to its non-regulatory cell counterpart are shown. (**d**) The percentage of total CTLA-4 expression is shown in SIV-uninfected (*n*=9) and chronically SIV-infected (*n*=8) rhesus macaques. The horizontal bars of each graph indicate the median value and are listed where appropriate for clarity. Statistical analyses were performed by Mann–Whitney (Wilcoxon) tests to compare unpaired, nonparametric values and significance is denoted by asterisks where *P<0.05, **P<0.01 and ***P<0.001.

To determine if T_FR_ are likewise increased during SIV infection, we analysed lymphoid cells from uninfected or SIVmac239-infected rhesus macaques by flow cytometry. As SIV infection reduces cell surface CD4, we gated on viable CD3^+^CD8^−^ cells, as previously described in humans^33^. The surface phenotype of CD25^hi^CD127^−^ has been shown in humans to encompass Tregs and allow for live cell sorting^34^,^35^,^36^. We validated that this phenotype accurately detects Tregs in SIV-uninfected and SIV-infected rhesus macaques (Fig. 2a). Here, Tregs were defined as viable CD3^+^CD8-CD25^hi^CD127^−^, whereas T_FR_ were defined as the subpopulation that are CXCR5^+^, and GC T_FR_ as the subpopulation that are CXCR5^hi^ and PD-1^hi^. In chronic SIV infection, percentages of Tregs, and particularly T_FR_ and GC T_FR_, were significantly increased compared with SIV-uninfected animals (Fig. 2b). We next determined the ratio of regulatory cell populations to their non-regulatory counterpart and found the ratios of T_FR_ to T_FH_ and GC T_FR_ to GC T_FH_ were significantly higher than those from SIV-uninfected animals, whereas the overall ratio of Treg/non-Treg CD3^+^CD8^−^ cells did not differ between infected and uninfected animals (Fig. 2c). Treg, T_FR_ and GC T_FR_ subsets also expressed significantly higher levels of CTLA-4 in SIV-infected compared with SIV-uninfected animals (Fig. 2d).

### T_FR_ expansion is mediated by HIV infection

**Figure 3:**
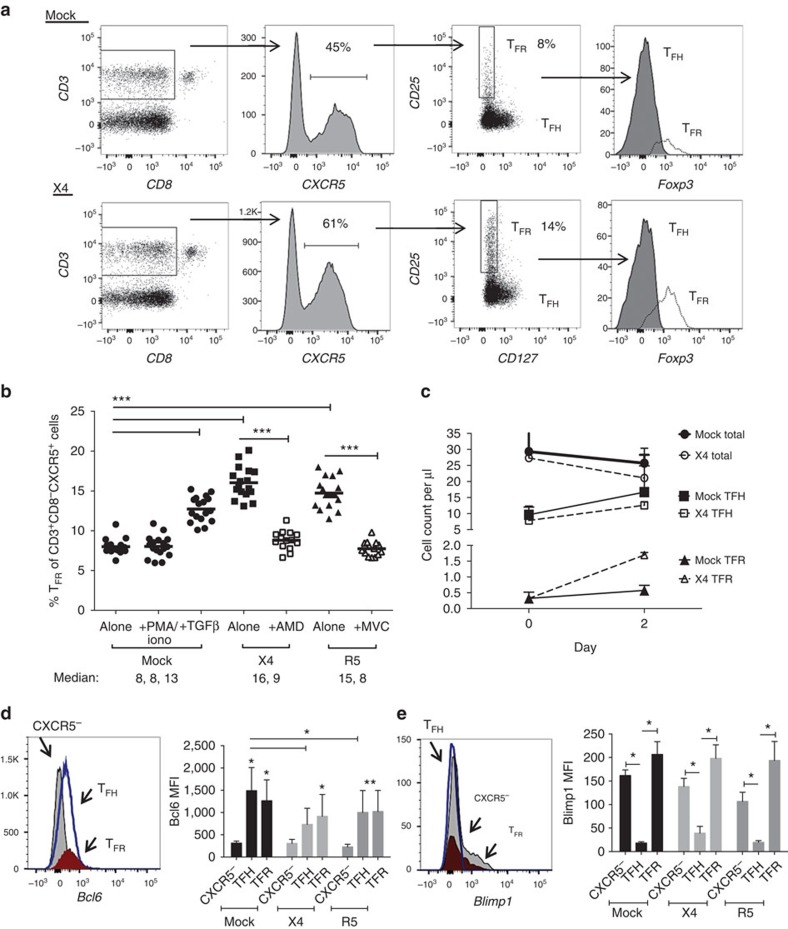
HIV entry and replication promote T_FR_ expansion. Disaggregated tonsil cells were spinoculated with X4 or R5 HIV and T_FR_ populations were analysed by flow cytometry (*n*=15). (**a**) A representative example of tonsil cell flow gating. From viable CD3^+^CD8^−^ cells, T_FR_ are defined as CXCR5^+^ and CD25^hi^CD127^–^. T_FR_ cells contain Foxp3^+^ cells, whereas remaining T_FH_ (CXCR5^+^CD25^lo/−^) cells are Foxp3^−^. (**b**) Percentages of T_FR_ determined by gating strategies in **a** are shown. Experimental conditions include mock-spinoculated cells cultured with PMA (50 ng ml^−1^) and ionomycin (1 μg ml^−1^) or exogenous TGF-β (100 ng ml^−1^) for 24 h and cells pretreated to block CXCR4 (AMD, 200 μM) and CCR5 (MVC, 2 μM). (**c**) Using flow cytometry counting beads, the number of cells per μl were determined for total (CD3^+^CD8^−^), T_FH_ (CXCR5^+^CD25^lo/−^) and T_FR_ (CXCR5^+^CD25^hi^CD127^−^) subsets in mock- and X4-spinoculated samples (*n*=3). (**d**) Bcl-6 expression is shown in CXCR5− (grey), T_FH_ (blue) and T_FR_ (red) populations after mock-, X4- or R5-spinoculation (*n*=5). (**e**) Blimp-1 expression was also determined as in **d**. The horizontal bars of each graph indicate the median value and are listed where appropriate for clarity. Statistical analyses were performed by Friedman nonparametric tests (**b**,**d**,**e**) and significance is denoted by asterisks where *P<0.05, **P<0.01 and ***P<0.001.

To characterize mechanisms underlying T_FR_ expansion in HIV/SIV infection, we utilized an *ex vivo* model of HIV-1 infection in human tonsil cells. Disaggregated tonsil cells were spinoculated with NL4-3 based X4- or R5-tropic green fluorescent protein (GFP) reporter viruses and cultured for 2 days. Productive infection was confirmed by the presence of GFP expression after 2 days and percentages of GFP^+^ T_FR_ ranged from 1 to 15%. Using the flow gating strategy described above for rhesus macaques, we demonstrated that CD25^hi^CD127^−^ cells encompassed the majority of Foxp3^+^ cells (Fig. 3a). Compared with mock-spinoculated tonsil cells, a 24-h stimulation of mock-spinoculated tonsil cells with phorbol 12-myristate 13-acetate (PMA) and ionomycin did not significantly increase T_FR_, whereas the positive control of exogenous TGF-β (ref. 37) did (Fig. 3b). A more marked expansion of T_FR_ was observed with either X4- or R5-spinoculation (Fig. 3b). Pretreatment of cells before spinoculation with chemokine co-receptor antagonists, bicyclam (AMD) for X4 and maraviroc (MVC) for R5, inhibited on average 80 and 86% of productive HIV infection, respectively. Both AMD and MVC prevented T_FR_ expansion (Fig. 3b). To determine if T_FR_ expansion represented true numerical increases, mock- and X4-spinoculated cultures were analysed using flow cytometry counting beads. Total CD3^+^CD8^−^ cells were decreased and T_FH_ cells were slightly lower 2 days after X4-spinoculation compared with mock-spinoculated cells (Fig. 3c). Conversely, the T_FR_ population was approximately threefold higher in X4-spinoculated cells compared with mock-spinoculated cells (Fig. 3c). We further confirmed the phenotype of T_FR_ by determining expression of the transcription factors Bcl-6 and Blimp-1, and evaluated the effects of *ex vivo* HIV-1 infection on their expression. Bcl-6 expression was significantly higher in both T_FH_ and T_FR_ compared with non-follicular (CXCR5^−^) populations in mock-spinoculated cells at day 2 (Fig. 3d). With X4 or R5 spinoculation, T_FH_ had significantly lower Bcl-6 expression compared with mock-spinoculated cells, whereas Bcl-6 expression in T_FR_ was not significantly altered by HIV spinoculation (Fig. 3d). Blimp-1 expression was significantly higher in non-follicular and T_FR_ populations compared with T_FH_ in mock-spinoculated cells at day 2, but was not significantly altered after X4 or R5 spinoculation (Fig. 3e).

### IDO and TGF-β production contribute to T_FR_ expansion

**Figure 4:**
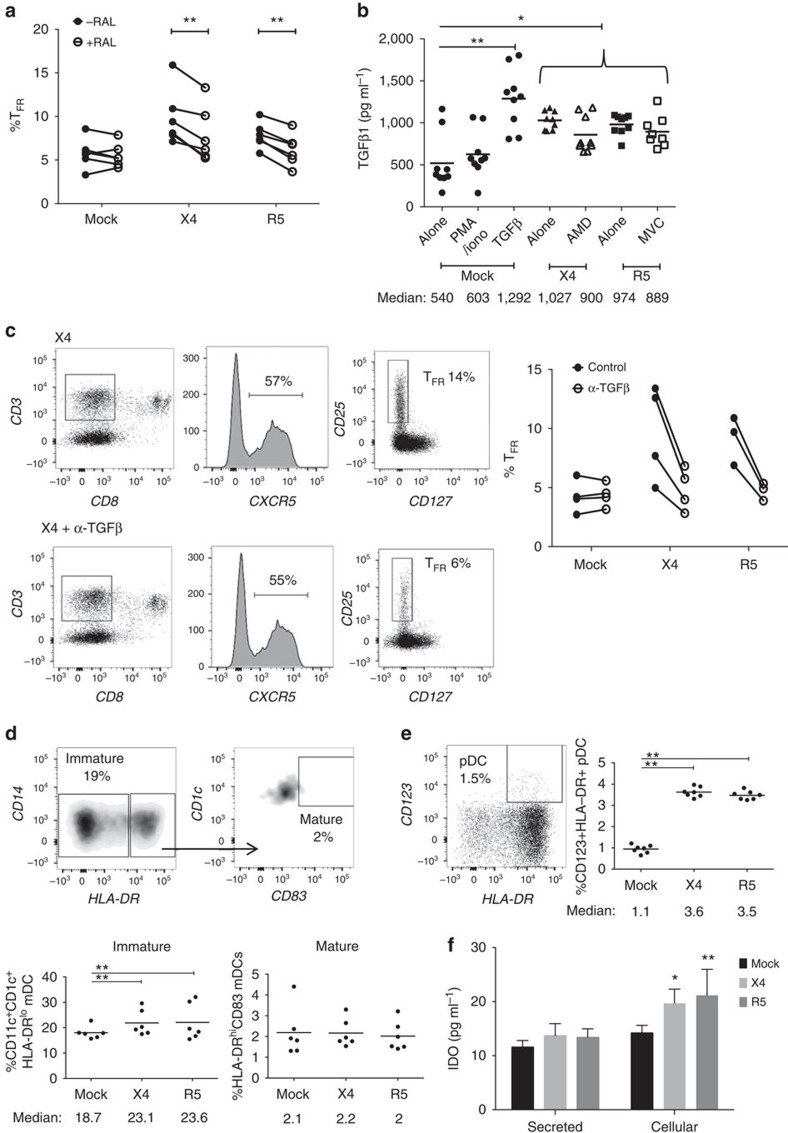
Viral replication, TGF-β signalling and regulatory dendritic cells promote T_FR_ expansion. Tonsil cells were mock-spinoculated or spinoculated with X4 or R5 virus and cultured for 2 days under a variety of conditions. (**a**) Tonsil cells were treated with the integrase inhibitor raltegravir (RAL, 10 μM) during culture to allow viral entry but prevent integration and percentages of T_FR_ determined (*n*=5). (**b**) Tonsil cells were cultured under the conditions shown and TGF-β-1 levels were measured in culture supernatant by ELISA (*n*=8). (**c**) Tonsil cells were cultured in the presence of TGF-β blocking antibodies (2 μg ml^−1^) and the percentages of T_FR_ were measured (*n*=4). Addition of anti-TGF-β antibodies did not influence cell viability. (**d**) Tonsil cells were cultured under the conditions shown and then analysed for the presence of immature myeloid DCs (CD11c^+^CD1c^+^HLA-DRlo) and mature myeloid DCs (CD11c^+^CD1c^+^HLA-DR^+^CD83^+^; *n*=6). (**e**) Samples in **d** were also analysed for activated plasmacytoid DCs (CD123^+^HLA-DR^+^, *n*=6). (**f**) Cell culture supernatants and lysates from **d**,**e** were analysed by ELISA to quantitate IDO production (*n*=6). The horizontal bars of each graph indicate the median value and are listed where appropriate for clarity. Statistical analyses were performed using Wilcoxon matched-pairs tests (**a**,**c**) or Mann-Whitney ranked sums tests (**b**,**d**–**f**) and significance is denoted by asterisks where *P<0.05, **P<0.01 and ***P<0.001.

To further address viral life cycle contribution to T_FR_ expansion, tonsil cells were treated with the integrase inhibitor raltegravir (RAL) after spinoculation. RAL blocked on average 91% of productive HIV infection and significantly decreased, but did not fully suppress, T_FR_ expansion (Fig. 4a). In addition, TGF-β is crucial for stable Foxp3 expression in Tregs, so culture supernatants were analysed for TGF-β production. Stimulation of mock-spinoculated cells with PMA and ionomycin did not lead to increased TGF-β production, whereas the presence of either X4 or R5 virus, even with AMD or MVC pretreatments before spinoculation, led to significant increases in TGF-β (Fig. 4b). When tonsil cells were cultured in the presence of TGF-β-neutralizing antibodies, T_FR_ expansion was inhibited in both X4- and R5-spinoculation (Fig. 4c).

Regulatory DC are described as myeloid and plasmacytoid DCs that have an immature or semi-mature phenotype and produce anti-inflammatory molecules such as indoleamine 2,3-dioxygenase (IDO) that generates Tregs^38^. It has previously been reported that regulatory DCs inhibit effector T-cell function by exhibiting a lack of co-stimulatory molecule expression^39^ and can produce IDO in lymphoid tissues during HIV and SIV infection^40^,^41^,^42^. In tonsil cultures, there were significantly more immature myeloid DCs with both X4- and R5-spinoculation, but no significant increase in mature myeloid DCs when compared with mock-spinoculated cells (Fig. 4d). X4- and R5-spinoculation also significantly increased activated plasmacytoid DC compared with mock spinoculation (Fig. 4e). Culture supernatants and lysed cells were then measured for IDO production. There was a significant increase in IDO with either X4- or R5-spinoculation compared with mock-spinoculation (Fig. 4f). We further analysed these culture supernatants for interferon (IFN)-γ production, as IFN signalling is upstream of IDO production. X4- and R5-spinoculated cultures tended to have higher levels of IFN-γ, but this increase was not statistically significant (Supplementary Fig. 2a).

### T_FR_ have high proliferation and low apoptosis rates

**Figure 5:**
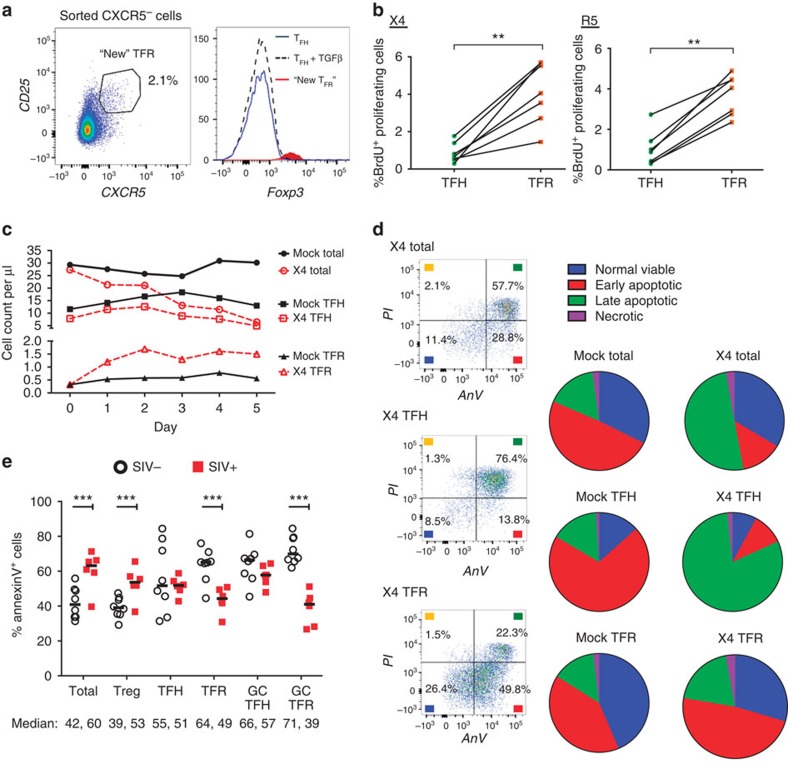
Acquisition of CXCR5, enhanced proliferation and reduced apoptosis promote T_FR_ expansion. (**a**) T_FH_ and CXCR5^−^ T-cell populations were sorted and cultured without stimulation, or T_FH_ were cultured in the presence of exogenous TGF-β (100 ng ml^−1^). CXCR5 expression was analysed on sorted CXCR5^−^ cells after 2 days and cells expressing CXCR5 are labelled as ‘new T_FR_’. The Foxp3 expression levels of T_FH_, T_FH_ cultured with TGF-β and ‘new’ T_FR_ were determined (*n*=3). (**b**) T_FH_ and T_FR_ were mock-, X4- or R5-spinoculated and cell proliferation measured by BrdU incorporation after 2 days of culture (*n*=7). (**c**,**d**) A 5-day time course was performed to monitor T-cell population counts and rates of cell death with mock- or X4-spinoculated tonsil cells (*n*=3). (**c**) Average counts of total (CD3^+^CD8^−^, circles), T_FH_ (CXCR5^+^CD25^lo/−^, squares) and T_FR_ (CXCR5^+^CD25^hi^CD127^−^, triangles) are shown for the duration of culture (*n*=3). (**d**) Stages of cell death are shown at day 5 and defined as early apoptosis (AnnexinV^+^), late or advanced apoptosis (AnnexinV^+^ PI^+^) and necrotic death (PI^+^; *n*=3). (**e**) Cell subsets from disaggregated lymphoid tissues of chronically SIV-infected (*n*=6) and uninfected rhesus macaques (*n*=8) were analysed for apoptosis by percent Annexin-V binding. Cell phenotypes were defined as total (CD3^+^CD8^−^), Treg (CD3^+^CD8-CD25^hi^CD127^−^), T_FH_ (CD3^+^CD8-CXCR5^+^CD25^lo/−^), T_FR_ (CD3^+^CD8-CXCR5^+^CD25^hi^CD127^−^), GC T_FH_ (CD3^+^CD8-CXCR5^+^PD1^hi^CD25^lo/−^) and GC T_FR_ (CD3^+^CD8-CXCR5^+^PD1^hi^CD25^hi^CD127^−^). Statistical analyses were performed by Wilcoxon matched-pairs tests (**b**) or Mann–Whitney tests (**e**) and significance is denoted by asterisks where **P<0.01 and ***P<0.001.

In mice, T_FR_ arise from conventional Tregs, rather than from T_FH_^15^. To investigate the origin of T_FR_ in human cells, we evaluated whether tonsil T_FH_ phenotypically converted to T_FR_ or whether Tregs gain CXCR5 expression. T_FH_ (CD3^+^CD8^−^CXCR5^+^CD25^−^) and non-follicular (CD3^+^CD8^−^CXCR5^−^) T-cell populations were sorted and cultured without HIV-spinoculation for 2 days. Interestingly, T_FH_ did not acquire Foxp3 expression, even in the presence of exogenous TGF-β, whereas a small population of cells from the initial CXCR5^−^ population acquired CXCR5 expression (Fig. 5a). These cells, labelled ‘new’ T_FR_, expressed high levels of Foxp3 (Fig. 5a).

We further hypothesized that cell proliferation may contribute to T_FR_ expansion. We utilized 5-bromodeoxyuridine (BrdU) labelling in tonsil cells to measure the relative rates of T_FR_ and T_FH_ proliferation. Tonsil cells were mock-, X4- or R5-spinoculated and BrdU DNA incorporation was measured after 2 days by flow cytometry. BrdU incorporation rates were low overall, but in the context of HIV infection, T_FR_ incorporated significantly more BrdU than T_FH_ (Fig. 5b).

To evaluate the duration of tonsil T_FR_ increases, we extended the counting experiments in Fig. 3c for 5 days. Total CD3^+^CD8^−^, T_FH_ and T_FR_ populations from mock-spinoculated samples remained numerically stable up to 5 days (Fig. 5c). In X4-spinoculated samples, total and T_FH_ cells declined by day 5, whereas T_FR_ were elevated at day 2 and remained so through day 5 (Fig. 5c). We also analysed cell death rates by Annexin-V and propidium iodide (PI) staining in mock- and X4-spinoculated tonsil cells. Total CD3^+^CD8^−^ cells and T_FH_ cells displayed increased rates of late apoptosis (Annexin^+^PI^+^) and necrosis (PI^+^) in X4-spinoculated samples compared with mock-spinoculated cells (Fig. 5d). The majority of T_FR_ remained in a state of viability or early apoptosis (Annexin^+^) in X4-spinoculated tonsil cells, similar to what was seen with mock-spinoculated cells (Fig. 5d). To determine if this occurs during *in vivo* infection, we analysed Annexin-V staining in disaggregated rhesus macaque lymphoid cells. The levels of Annexin-V staining in total CD3^+^CD8^−^ and Treg populations were significantly higher in SIV-infected animals compared with SIV-uninfected animals (Fig. 5e). There was not a significant difference in the level of Annexin-V staining in T_FH_ cells in SIV-infected compared with SIV-uninfected animals (Fig. 5e). In contrast, the levels of Annexin-V staining in T_FR_ and GC T_FR_ were significantly lower in SIV-infected animals compared with SIV-uninfected animals (Fig. 5e).

### T_FR_ regulatory phenotypes in HIV infection *ex vivo*

**Figure 6:**
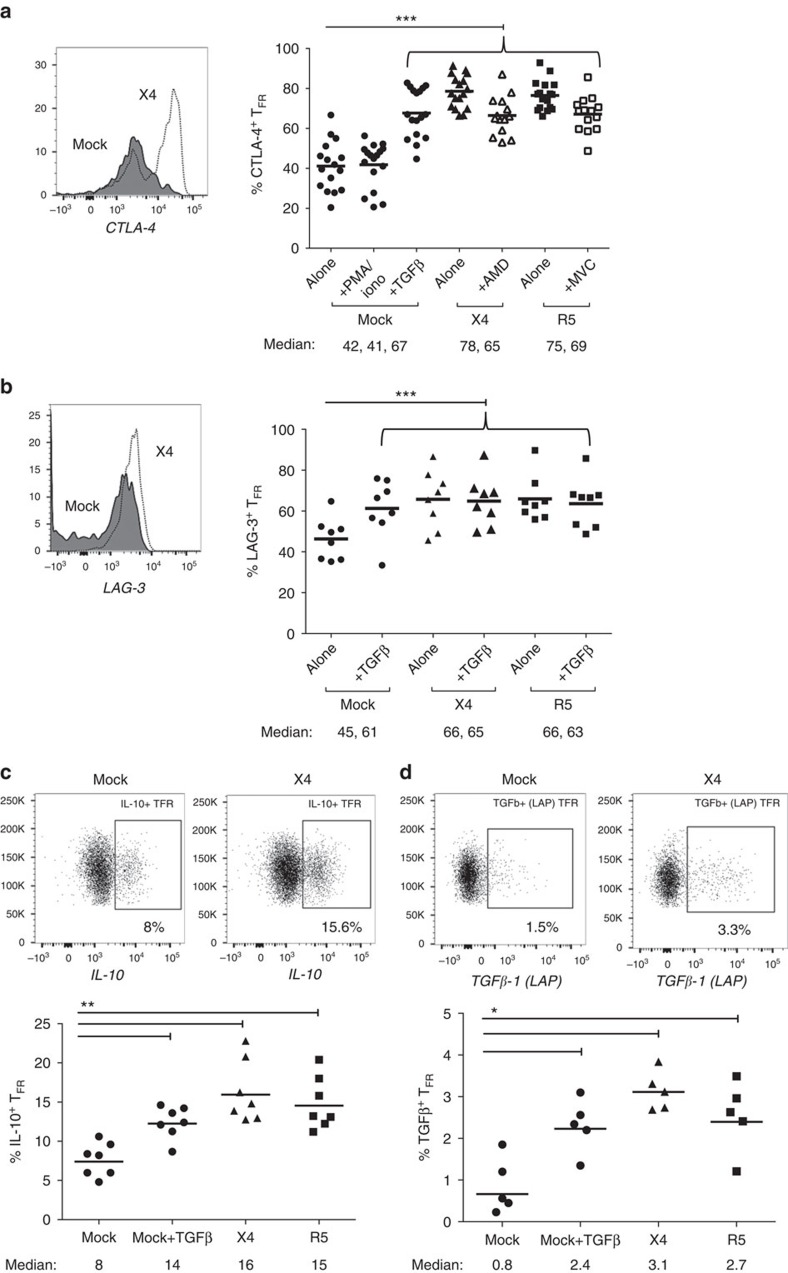
T_FR_ exhibit an enhanced regulatory phenotype in *ex vivo* HIV infection. Tonsil cells were mock-, X4-, or R5-spinoculated and cultured under experimental conditions as indicated. T_FR_ were then analysed for expression of regulatory receptors and cytokine production by intracellular cytokine staining. (**a**) Percentage of total (surface and intracellular) T_FR_ CTLA-4 expression (*n*=15). (**b**) Percentage of surface T_FR_ LAG-3 expression (*n*=8). (**c**) Production of IL-10 by T_FR_ (*n*=7). (**d**) Production of TGF-β-1 (measured as LAP) by T_FR_ (*n*=5). The horizontal bars of each graph indicate the median value and are listed where appropriate for clarity. Statistical analyses were performed by Friedman (**a**,**b**) or Mann–Whitney tests (**c**,**d**) and significance is denoted by asterisks where *P<0.05, **P<0.01 and ***P<0.001.

We next sought to determine the regulatory phenotype of tonsil T_FR_ during infection. CTLA-4 and LAG-3 are used to contact and inhibit effector cell function, GITR is expressed on activated and proliferating cells, whereas galectin receptors are an important family in promoting effector cell apoptosis^43^. Both X4- and R5-spinoculation caused T_FR_ to significantly upregulate total CTLA-4 (Fig. 6a) and LAG-3 (Fig. 6b), as well as GITR and galectin-3, whereas galectin-9 expression was not altered (Supplementary Fig. 2b). Regulation of T_FH_ has previously shown to occur through PD-1 ligation^13^, however, PD-L1 was expressed on fewer than 3% of T_FR_ following either mock or X4 spinoculation (*n*=3). Blockade of chemokine co-receptors partially inhibited CTLA-4 and GITR upregulation on T_FR_ (Fig. 6a and Supplementary Fig. 2a). Blockade of CD4 before spinoculation inhibited upregulation of CTLA-4 (Supplementary Fig. 3a) and GITR (Supplementary Fig. 3b) during infection. Blockade of TGF-β led to downregulation of CTLA-4 on T_FR_ during infection (Supplementary Fig. 3c).

IL-10 regulates effector cell function, whereas TGF-β prevents inflammatory responses and promotes Foxp3 expression^43^. IL-10 and TGF-β (LAP-1) production by tonsil T_FR_ was determined by intracellular cytokine staining. Mock-spinoculated cultures treated with exogenous TGF-β as a positive control showed a significant increase of IL-10^+^ T_FR_ (Fig. 6c). X4- and R5-spinoculation led to significant increases of IL-10^+^ T_FR_, which tended to be higher than TGF-β-treated T_FR_ (Fig. 6c). Similar trends were observed in regards to TGF-β^+^ T_FR_ (Fig. 6d). In addition, analysis of culture supernatant showed that infection significantly upregulated IL-10 compared with mock-spinoculated controls (Supplementary Fig. 2c), whereas the presence of X4 or R5 virus did not significantly increase IL-35 production (Supplementary Fig. 2d).

### T_FR_ dysregulate T_FH_ activity

**Figure 7:**
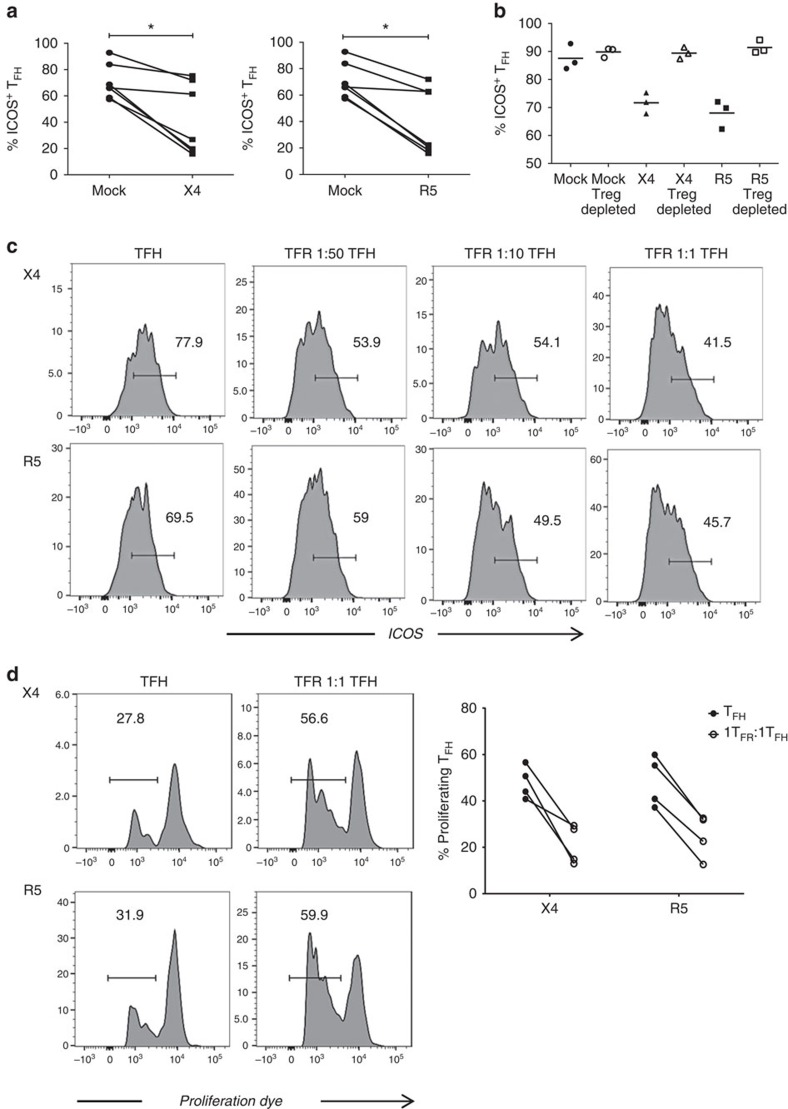
T_FR_ impair T_FH_ ICOS expression and proliferation. (**a**) Tonsil T_FH_ were analysed for surface ICOS, following mock-, X4-, or R5-spinoculation (*n*=7). (**b**) CD25^+^ regulatory cells were depleted from each culture condition and ICOS expression on T_FH_ was analysed (*n*=3). (**c**) Tonsil T_FH_ and T_FR_ were isolated, spinoculated and co-cultured for 2 days at the indicated ratios. Representative images of ICOS expression on T_FH_ surface are shown (*n*=2). (**d**) Tonsil T_FH_ were isolated, labelled with eFluor 670 proliferation dye, and cultured alone or with an equal number of T_FR_. Proliferation was measured by dye dilution (*n*=4). The horizontal bars of each graph indicate the median value and are listed where appropriate for clarity. Statistical analyses were performed using Friedman nonparametric tests (**a**) and significance is denoted by asterisks where *P<0.05.

T_FR_ have been shown to modulate T_FH_ function and thereby control GC reactions in mice^14^,^15^,^16^. Given that T_FH_ exhibit impaired function in chronic HIV infection^13^ we investigated whether T_FH_ were impaired by T_FR_ in the tonsil model. We first compared the expression of ICOS, a key molecule for T_FH_ maintenance and function, in X4- or R5-spinoculation to mock-spinoculation. With both X4- and R5-spinoculation, T_FH_ demonstrated a significant reduction of ICOS expression (Fig. 7a). We evaluated the role of Tregs in T_FH_ ICOS downregulation by depleting CD25^+^ regulatory cells, which effectively removes all Foxp3^+^ cells from tonsil cell culture, and analysing T_FH_ after 2 days. ICOS expression on T_FH_ was high in mock-spinoculated controls regardless of the presence or absence of Treg (Fig. 7b). With X4- or R5-spinoculation, however, ICOS expression was not downregulated when Tregs were absent (Fig. 7b). Although CD25^+^ cell depletion effectively removes overall regulatory populations, we could not rule out that T_FR_ may have functions differing from those of Tregs. We therefore examined the regulatory effects of T_FR_ specifically by isolating tonsil T_FH_ and adding T_FR_ back to T_FH_ at increasing ratios. In X4- and R5-spinoculated cultures, we observed that T_FH_ ICOS levels were decreased as the ratio of T_FR_ increased (Fig. 7c).

Previous studies using *ex vivo* suppression assays have shown that Treg inhibit CD4^+^ T-cell proliferation and cytokine production in HIV infection^18^,^21^. First, we investigated the ability of T_FR_ to suppress proliferation of T_FH_ in the context of *ex vivo* HIV infection. Tonsil T_FH_ and T_FR_ were isolated, spinoculated with either X4 or R5 virus, and cultured either alone or with equal numbers of T_FR_ for 4 days in the presence of anti-CD3/anti-CD28 antibodies and IL-2. T_FR_ consistently inhibited proliferation of T_FH_ in both X4- and R5-spinoculated cultures (Fig. 7d).

**Figure 8:**
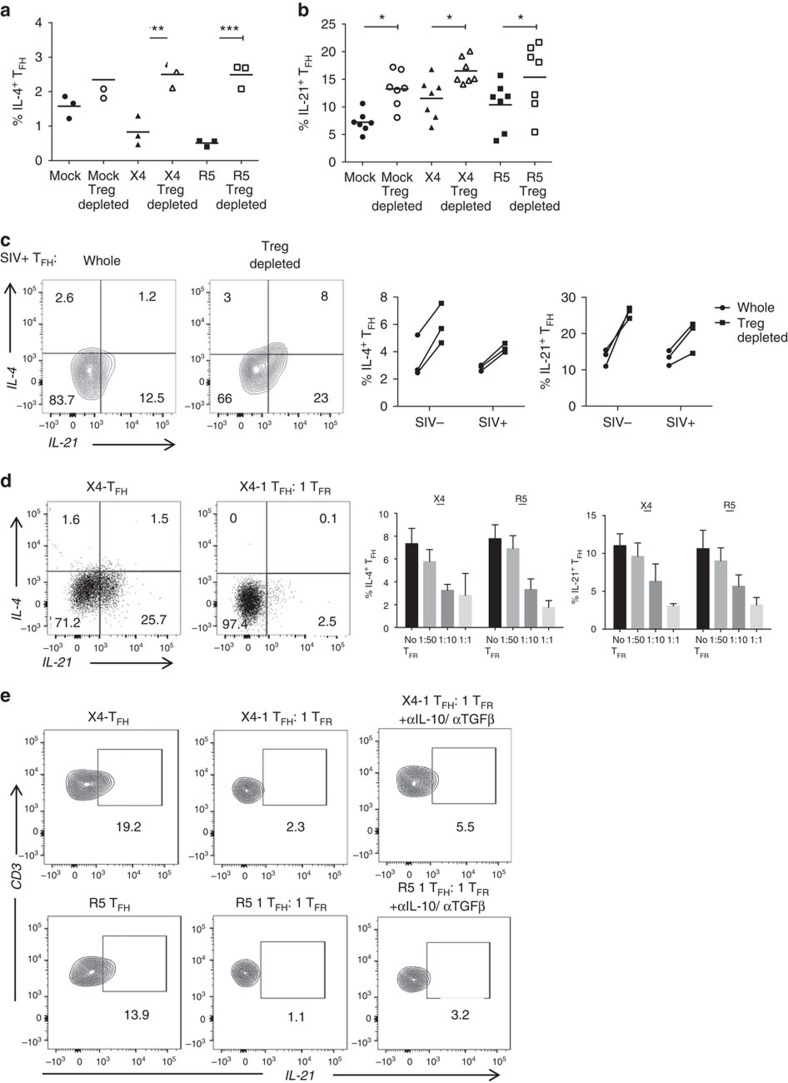
T_FR_ impair T_FH_ cytokine production. (**a**) Tonsil T_FH_ were analysed by intracellular cytokine staining for IL-4 and IL-21 production following mock-, X4- or R5-spinoculation. (**b**) CD25^+^ regulatory cells were depleted before spinoculation and IL-4 (*n*=3) and IL-21 (*n*=7) production by T_FH_ cells were analysed after 2 days. (**c**) IL-4 and IL-21 production by T_FH_ were analysed in disaggregated lymph node cells from rhesus macaques with and without CD25-depletion (*n*=3). (**d**) Sorted populations of tonsil T_FH_ and T_FR_ were spinoculated, T_FR_ were added back to T_FH_ at an increasing ratio, and T_FH_ production of IL-4 and IL-21 were measured by intracellular cytokine assays at day 2 (*n*=4). (**e**) Sorted tonsil T_FH_ and T_FR_ were cultured for 2 days in the presence of IL-10- and TGF-β-neutralizing antibodies and T_FH_ production of IL-21 was analysed (*n*=2). Statistical analyses were performed by Friedman nonparametric tests (**a**,**b**) and significance is denoted by asterisks where *P<0.05 and ***P<0.001.

Next, we investigated the role of T_FR_ on T_FH_ cytokine production. Initially, we observed that tonsil T_FH_ display decreased IL-4 production in either X4- or R5-spinoculated cultures, whereas IL-21 production did not have a uniform trend (Supplementary Fig. 4a). Removal of CD25^+^ Tregs led to significant increases in IL-4 (Fig. 8a) and IL-21 (Fig. 8b) production by tonsil T_FH_ following mock-, X4- and R5-spinoculation and significant increases in IL-4 and IL-21 production by T_FH_ in cells from both uninfected and chronically SIV-infected animals (Fig. 8c). We further validated these observations by co-culturing tonsil T_FH_ with increasing ratios of T_FR_ following spinoculation with X4 virus. The absence of T_FR_ led to increased T_FH_ production of IL-4 and IL-21 (Fig. 8d). The regulation of T_FH_ was T_FR_ dose-dependent, as an increasing ratio of T_FR_ led to increasing inhibition of T_FH_ IL-4 and IL-21 production (Fig. 8d). To determine if the suppressive capacity of T_FR_ was due to the production of suppressive cytokines, we performed intracellular cytokine assays in the presence of both IL-10- and TGF-β-neutralizing antibodies. Previous reports show that IL-10 potently blocks IL-4 production^44^, so we specifically addressed IL-21 production by T_FH_. Neutralization of IL-10 and TGF-β restored some, but not the majority of IL-21 production by T_FH_ in the presence of T_FR_ (Fig. 8e).

## Discussion

In the present study, we provide a comprehensive analysis of T_FR_ in HIV and SIV infection. We show that T_FR_ are expanded in secondary lymphoid tissues during both chronic HIV and SIV infection. In addition, we utilize HIV infection *ex vivo* to show that cell proliferation, regulatory DCs, TGF-β signalling, Treg acquisition of CXCR5 and resistance to apoptosis play a role in T_FR_ expansion. Finally, we demonstrate that T_FR_ are able to inhibit proliferation, ICOS expression and IL-4 and IL-21 production by T_FH_. Collectively, these findings reveal that T_FR_ play a crucial role in the impairment of T_FH_ during HIV and SIV infection.

Although Tregs are diminished in gut-associated lymphoid tissue during HIV infection^23^, prior studies indicate an increase in Tregs in LNs^21^,^26^,^45^. We found that absolute numbers of CD4^+^Foxp3^+^ cells in follicles and GCs per average LN cross-section were increased in HIV-infected individuals compared with uninfected individuals. Furthermore, following *ex vivo* infection of human tonsil cells, we demonstrated that T_FR_ increase both proportionally and numerically. As Foxp3 can be transiently upregulated after T-cell activation, we evaluated whether percentages of activated cells were related to Foxp3 expression. Nevertheless, we found no correlation between percentages of activated LN cells and Tregs and T_FR_, and numbers of GC T_FR_ were inversely related to percentages of HLA-DR^+^CD38^+^CD4^+^ cells, which is the opposite of what would expect if immune activation was driving Foxp3 expression.

Blockade of the HIV chemokine co-receptors CXCR4 and CCR5 prevented T_FR_ expansion in tonsil cells, thus demonstrating that viral entry was necessary to promote expansion. Intriguingly, however, treatment with the integrase inhibitor RAL, which blocks the virus replication cycle after cell entry, largely blunted but did not completely ablate T_FR_ expansion. It has recently been demonstrated that many cells during *ex vivo* HIV infection are abortively infected and only a minority of infected cells are productively infected^46^. Viral sensing mechanisms are important for innate immune cell activation in HIV infection and similar mechanisms may also contribute to T_FR_ activation and proliferation. HIV RNA sensing through internal TLR7 activates pDC^47^ and TLR7 ligation has been shown to induce CD4 T-cell proliferation^48^. It is possible that HIV sensing by T_FH_ or T_FR_ contributes to T_FR_ expansion and further studies concerning abortive and productive infections are warranted. It has been reported that Treg increases in untreated HIV-infected individuals are diminished after treatment with antiretroviral therapy (ART), but do not normalize^49^. A recent study demonstrated that addition of MVC to ART regimens led to further reductions in Tregs in individuals who were already virologically suppressed^50^. These data, in conjunction with our findings, suggest that in HIV-treated individuals who are fully suppressed on ART regimens that do not include MVC, ongoing nonproductive HIV infection or a low level of HIV replication is occurring resulting in persistent T_FR_ expansions that may contribute to immune impairments.

Tonsil T_FR_ increased their regulatory capacity during X4 or R5 HIV infection, as evidenced by increased CTLA-4, LAG-3, GITR and galectin-3 expression and production of IL-10 and TGF-β. In contrast to T_FR_ expansions, however, the increased regulatory phenotype was only partially reduced by the addition of AMD and MVC, but fully reduced by CD4 blockade. This finding is consistent with the observation that HIV binding to CD4 molecules enhances their regulatory phenotype and suppressive capabilities^31^. In addition, we found that blockade of TGF-β signalling fully inhibited T_FR_ regulatory phenotype enhancement during HIV infection. Importantly, we found almost no expression of PD-L1 by tonsil T_FR_, suggesting that this regulatory molecule does not mediate their effects on T_FH_. Dissecting out the distinct mechanisms responsible for expansion and enhanced regulatory profiles of T_FR_ in HIV infection could identify potential therapeutic targets to suppress excessive T_FR_ activity and could have relevance to host–pathogen interactions in other diseases as well.

We also investigated whether intrinsic characteristics of T_FR_ might promote their expansion during HIV infection. We found significant increases in cell proliferation of tonsil T_FR_ compared with tonsil T_FH_ during both X4 and R5 HIV infection. It has been well-established that regulatory DCs induce Tregs in HIV infection, and exert this effect in part by low rates of maturation and co-stimulatory molecules^39^ and in part by IDO production^40^,^51^. The lack of DC maturation observed during HIV infection could contribute to increased Treg and T_FR_^52^. In tonsil cells, we found that myeloid DCs were mostly immature, whereas activated plasmacytoid DCs were significantly increased in HIV infection, which recapitulates previous studies in HIV^39^,^53^,^54^ and SIV^41^,^42^ infection *in vivo*. Tonsil cell cultures also exhibited increased levels of IDO, the IDO upstream activator IFN-γ and TGF-β during HIV infection. Type I IFNs were not specifically studied in this work, but they have also shown to be increased in tonsil cells infected with NL4-3 HIV^55^. These factors present in the follicular milieu likely contribute to enhanced proliferation and numerical increases of T_FR_.

HIV-infected individuals have high rates of overall CD4^+^ T-cell apoptosis^56^ and higher levels of Annexin-V binding and caspase-3 activation in Tregs compared with uninfected controls^29^. We similarly found that total CD3^+^CD8^−^ cells and Tregs had higher levels of Annexin-V binding in chronically SIV-infected rhesus macaques. Remarkably, however, we found that T_FR_, GC T_FR_ and GC T_FH_ had lower levels of Annexin-V binding in SIV-infected rhesus macaques compared with uninfected animals. Furthermore, T_FR_ and GC T_FR_ had the lowest levels of Annexin-V binding of any subset in SIV-infected macaques, but not in uninfected macaques. These findings imply that regulatory follicular cells may be more resistant to apoptosis than other subsets, which could promote their accumulation. This could explain our observation that lymphoid tissue ratios of T_FR_ to T_FH_ and GC T_FR_ to GC T_FH_ in chronically SIV-infected rhesus macaques were significantly higher than those from SIV-uninfected animals, but that this phenomenon was not observed in the Treg population as a whole compared with non-Treg CD3^+^CD8^−^ cells. We recently reported that tonsil follicular CD4^+^ T cells have elevated Bcl-2 expression and Bcl-2 is further upregulated on cells that are productively infected with R5 virus^57^. However, these studies did not discriminate between T_FR_ and T_FH_ populations. Thus, elevated Bcl-2 expression on T_FR_ could be a mechanism that protects T_FR_ from apoptosis. The mechanisms of T_FR_ apoptosis and how they contribute to T_FR_ number warrant further investigation.

In characterizations of T_FR_ in mice, it was shown that T_FR_ arise from non-T_FH_ precursors and express both Blimp-1 and Bcl-6 (refs 14, 15). To assess this in humans, we isolated both T_FH_ and non-follicular (CXCR5^−^) subsets from tonsils and looked for alterations in their phenotype. T_FH_ did not acquire a T_FR_ phenotype, even when provided with exogenous TGF-β. A small population of CXCR5^−^ cells, however, acquired CXCR5 expression and expressed Foxp3. Despite being numerically small, this new T_FR_ population could contribute substantially to increase the T_FR_ population. Intriguingly, it was recently shown that T_FH_ differentiation and maintenance was optimized by TGF-β in combination with T_FH_ cytokines IL-12 and IL-23, but naïve CD4^+^ T cells cultured with TGF-β alone acquired Treg expression profiles while promoting CXCR5 expression^58^. This further supports the notion that the expansions of T_FR_ observed in our study likely originate from non-T_FH_ precursors. We also find that human T_FR_ express Bcl-6 and Blimp-1 and neither was altered after HIV spinoculation. However, expression of Bcl-6 by T_FH_ was significantly reduced after HIV spinoculation. A more detailed analysis would be valuable to reveal the effects of HIV on both T_FH_ and T_FR_ transcriptional profiles.

A hallmark of HIV infection is the production of high levels of HIV-specific antibodies that are unable to suppress HIV^27^. HIV-specific neutralizing antibodies are produced in many individuals during HIV infection^59^, however, the virus is able to rapidly mutate and avoid recognition^60^,^61^. Although broadly neutralizing antibodies (bNabs) are effective in suppressing HIV replication^62^, few people develop bNabs^63^. Whether T_FR_ limit the ability of HIV-infected individuals to develop bNabs is an important question. BNabs are characterized by high-affinity maturation rates in GCs, and functional T_FH_ are crucial for somatic hypermutation levels of maturing B cells in follicles^6^. The level of bNabs in a cohort of HIV seronegative individuals who received an HIV vaccine positively correlated to functional circulating resting memory T_FH_^64^. We demonstrate that T_FR_ inhibit the ability of T_FH_ to proliferate, to produce crucial B-cell help cytokines IL-4 and IL-21, and maintain ICOS expression. Whether these factors are key to developing bNabs is of critical importance to address. Apart from direct effects on T_FH_, T_FR_ may inhibit bNab production through direct interactions with B cells, or indirectly by altering the follicle microenvironment or follicular DC network during HIV infection. Thus, understanding the impact of T_FR_ on the GC reaction and development of bNabs is complex. These questions cannot be readily addressed within tissue cultures systems, but would be best answered in an interventional study in which T_FR_ are manipulated.

The dynamics of GC formation in acutely SIV-infected rhesus macaques were recently characterized^65^. Interestingly, animals with the most impaired GC development experienced the most rapid disease progression. It would be important to evaluate whether a robust T_FR_ response correlates with the greatest impairment in GC formation in these animals. Interestingly, Treg-depleted mice infected with influenza had limited GC size and compromised influenza-specific T_FH_ responses^66^. One possible explanation is that T_FR_ are critical for GC formation and fine-tuning of immune responses during acute infections, but may exert more suppressive responses in chronic disease^67^. It will be important to determine the precise role of T_FR_ in GC development and maintenance during different stages of HIV, as well as in other chronic infections.

T_FR_ are emerging as important drivers of GC dynamics. Our findings that T_FR_ are expanded during HIV infection and impair T_FH_ function have multiple important implications. First, they provide a potential mechanism for the impaired humoral responses observed in untreated HIV-infected individuals, particularly the inability to develop bNabs. Second, they provide a potential explanation for persistent impairments in humoral immunity seen in HIV-infected individuals, despite good virologic suppression on ART that does not contain a CCR5 receptor blocker. They further suggest that prophylactic HIV vaccines should be designed to minimize induction of T_FR_, as they could impair development of protective antibody responses. Finally, these studies have implications for diseases and vaccine strategies apart from HIV infection, as it is likely that T_FR_ play a key role in immune responses to many infections. Thus, a better understanding of the role of T_FR_ in the context of HIV infection can provide important insights into the dynamics of GC responses and humoral immunity during infection and adds a new dimension to the intricacies of vaccine development.

## Methods

### Human and rhesus macaque subjects and clinical specimens

Inguinal LNs were obtained by excisional biopsy in an outpatient procedure as previously described^4^ from individuals with documented HIV-1 infection for at least 6 months, who were not receiving ART and had CD4^+^ T-cell counts of ≥300 per mm^3^. None of these subjects had an opportunistic infection, malignancy or acute illness at the time of LN excision. CD4^+^ T-cell counts ranged from 214 to 1,103 cells per mm^3^ (median, 478 cells per mm^3^), and plasma viral load ranged from 2.87 to 5.88 log_10_ copies per ml (median, 4.26 log_10_ copies per ml). A total of 10 (53%) of subjects were females, ages ranged from 21 to 50 years (median, 34 years) and 10 (53%) were white, including 3 Hispanics, and 9 were black (47%). Inguinal LNs were also obtained from HIV-1-seronegative individuals while they underwent a non-emergent surgical procedure in the groin area. A total of 6 (75%) of seronegative subjects were female, ages ranged from 32 to 71 years (median, 50 years), and 7 (88%) were white, including 1 Hispanic, and 1 (12%) was black. Informed consent was obtained from all LN donors. Human tonsils were obtained from the Colorado Children’s Hospital (Aurora, CO, USA) following routine tonsillectomy from individuals at low risk for HIV infection. Use of tonsil specimens for these studies was reviewed by the Colorado Multiple Institutional Review Board and determined to not constitute human subjects research, in accordance with guidelines issued by the Office of Human Research Protections (http://www.hhs.gov/ohrp/policy/checklists/decisioncharts.html), and consequently, informed consent was not required. All research involving human subjects conformed to the principles set forth in the Declaration of Helsinki and was approved by the Colorado Multiple Institutional Review Board. LNs and the spleen were obtained from SIVmac239-infected and uninfected Indian rhesus macaques (*Macaca mulatta*). Animals were infected either intravenously or intrarectally with SIVmac239, and had been infected from 12 to 241 weeks (median, 19.5 weeks) at the time that specimens were obtained. Plasma SIV RNA concentrations ranged from 3.77 to 6.76 log_10_ copies per ml (median, 5.72 log_10_ copies per ml), and CD4^+^ T cell counts ranged from 142 to 569 cells per mm^3^ (median, 348 cells per mm^3^). Of SIV-infected animals, 4 (40%) were female and they ranged in age from 7 to 17 years (median, 9 years). Of SIV uninfected animals, 2 (22%) were female and they ranged in age from 3 to 23 years (median, 4 years). Animals were housed and cared for in accordance with the American Association for Accreditation of Laboratory Animal Care standards in accredited facilities, and all animal procedures were performed according to protocols approved by the Institutional Animal Care and Use Committees of the Wisconsin National Primate Research Center. Tissues were either shipped overnight on ice in cold RPMI 1640 and disaggregated or disaggregated at the Wisconsin National Primate Research Center and frozen cells shipped on liquid nitrogen to the University of Colorado. Sample sizes in this study were based on availability of specimens and prior experience of the necessary sample size to see anticipated effects.

### Quantification of Foxp3^+^CD4^+^ cells in lymphoid tissues

Six-micrometre frozen sections of LN were thaw mounted onto slides and fixed in 1% paraformaldehyde (Sigma) in PBS. Two or three sections, at least 60 μm apart, were stained and evaluated for each subject. Indirect immunofluorescent staining was performed by first staining with Rabbit anti-IgD (Novus, cat# NB120–17184, 1:50) and mouse anti-Foxp3 (clone 236A/E7; eBiosciences, cat#14-4777-82, 1:100) for 1 h followed by detection with Pacific Blue anti-Rabbit IgG (cat# P10994, 1:100) and AF488 anti-mouse IgG (cat#A21200, 1:200; both from Invitrogen) for 30 min. After washing, sections were incubated for an additional hour with Rabbit anti-CD20 (Abcam, cat# ab27093, 1:400) and Rat anti-CD4 (clone YNB46.1.8; AbD Serotec, cat# MCA484G, 1:100) followed by detection with AF647 anti-rabbit IgG (Invitrogen, cat#A21443, 1:200) and AF594 anti-rat IgG (Invitrogen, cat#A21209, 1:200). Slides were covered with coverslips using SlowFade Gold (Invitrogen). Images of full sections were generated at × 60 using an Olympus VS120 scanner outfitted with an OrcaR2 camera (Olympus). Ten random images were extracted for each section from the main scans and Foxp3^+^CD4^+^ cells per mm^2^ of total, follicle (CD20^+^) and GC (CD20^+^IgD^−^) was determined using visual inspection and quantitative image analysis (Qwin Pro 3.4.0, Leica Microsystems). The areas of each LN cross-section, as well as the follicular and GC compartments, were determined by quantitative image analysis and averaged for each subject. The average number of Foxp3^+^CD4^+^ cells per LN cross-section and compartment was calculated by multiplying the frequency of Foxp3^+^CD4^+^ cells by the average area of the LN, follicle or GC in each subject.

### Quantification of activated CD4^+^ T cells in human LNs

Disaggregated LN cells (5 × 10^6^) were stained with antibodies to CD3-PEcy5-UCHT1 (BD Biosciences, 555334), CD4-APC-H7-RPAT4 (BD, 560158), CD38-FITC-240742 (Invitrogen, FAB2404F) and HLA-DR-APC-L243 (BD, 340549), evaluated by flow cytometry (LSR II, BD Immunocytometry Systems) and analysed using FlowJo (Tree Star). All antibodies were used at one test per 10^6^ cells.

### HIV reporter viruses and tonsil infection model

The HIV-1 NL4-3-based CXCR4 (X4)-tropic GFP reporter virus NLENG1-IRES^68^ and the CCR5 (R5)-tropic GFP reporter virus NLYUV3-GFP^33^ were used for tonsil cell infections. Virus stocks were prepared by transfecting 293T cells with either X4 or R5 plasmid constructs (Effectine, Qiagen) in complete DMEM (DMEM+10% FBS, penicillin/streptomycin and non-essential amino acids), collecting supernatants and spinning at 800*g* to remove debris. Viral stocks were stored at −80 °C before use. After disaggregation, 5 × 10^6^ tonsil cells were spinoculated with either GFP reporter virus or a mock spinoculation with an equal volume of complete DMEM for 2 h at 1,200*g* at room temperature. Cells were washed to remove unbound virus and media, and cultured for 2 days at 37 °C with 5% CO_2_ in RPMI with 10% FBS, L-glutamine and penicillin/streptomycin (R10) at a density of 1.5 × 10^6^ cells per ml. To block viral entry, tonsil cells were incubated in R10 at 37 °C with 5% CO_2_ for 1 h with 2 μM MVC, 200 μM bicyclam or 10 ng ml^−1^ of anti-human CD4 (BioLegend, 300515). To block HIV integration, tonsil cells were incubated with 10 μM of the integrase inhibitor RAL in R10 for 1 h before spinoculation and included in the culture for 2 days. To block TGF-β signalling, 2 μg ml^−1^ of anti-human TGF-β (BioLegend, 521703) was included for the duration of the culture. Exogenous TGF-β (Sigma, T7039) was added at a concentration of 100 ng ml^−1^ to induce Treg and T_FR_ in selected cultures as a positive control. At the end of culture, the cell supernatants were collected, centrifuged at 6,800*g* to remove debris and frozen at −80 °C for subsequent ELISA analysis. Cells were collected and immediately processed for analysis by flow cytometry.

### Flow cytometry analysis of T cells and DCs

Cells were blocked for 20 min with 2% BSA in PBS at 4 °C and then stained for 30 min at 4 °C in the dark. For T-cell phenotyping, the following anti-human-conjugated antibodies were used: CD3-APCCy7-UCHT1 (Tonbo, 25-0038), CD8-eVolve605-RPA-T8 (eBioscience, 83-0088), CD25-PECy7-BC96 (Tonbo, 60-0259), CXCR5-PE-MU5UBEE (eBioscience, 18–9185), CD127-Pacific Blue-A1095 (BioLegend, 351306), ICOS-ISA-3 (PECy7 or APC; BioLegend, 313520, 313510), PD-1-APC-MIH4 (eBioscience, 17–9969) or PD-1-APC-EH12.2H7 (BioLegend, 329908), Bcl6-PerCPCy5.5 −K112-91 (BD, 562198), Blimp1–646702 (R&D IC36081A), CTLA-4-PerCPeFluor710-14D3 (eBioscience, 46–1529), GITR-PE-eBioAITR (eBioscience, 12–5875), LAG-3-APC-3DS223H (eBioscience, 17–2239), Galectin-3− AF647-M3/38 (BioLegend, 125408) and Galectin-9− PerCPCy5.5–9M1–3 (BioLegend, 348910). The same panel was used for rhesus macaque cell staining with the single exception of CD3-APCCy7-SP34-2 (BD, 557757). Foxp3 expression in CD25^+^CD127^−^ cells was confirmed using Foxp3-PE-PHC1O1 and the Foxp3/Transcription factor permeabilization kit (eBioscience, 72–5776) according to the manufacturers’ protocols. For DC phenotyping, the following anti-human-conjugated antibodies were used: CD14-eFluor450-61D3 (eBioscience, 8048-0149), CD1c-PECy7-L161 (eBioscience, 25-0015), CD3-APCCy7-UCHT1 (Tonbo, 25-0038), CD11c-PE-3.9 (eBioscience, 12-0116), CD19-AF700-HIB19 (BioLegend, 302226), CD83-BV510-HB15e (BD, 563223), CD123-BV605-7G3 (BD, 564197), DC-SIGN-PerCPCy5.5-eB-h209 (eBioscience, 45–2099) and HLA-DR-APC-L243 (BD, 340691). All analyses were performed on Aqua LIVE/DEAD (Molecular Probes, L34957) or Ghost Dye 510 (Tonbo, 13–0870) negative cells. Fresh human tonsil cells were typically 70–90% viable after culture and cryopreserved rhesus macaque cells ranged from 40 to 80% viability after freeze/thaw. All antibodies were used at one test per 10^6^ cells. Cells were fixed with 2% paraformaldehyde. Data were acquired on a custom LSR II flow cytometer (Serial # H47100196, BD Immunocytometry System) with BDFACS Diva (v6.1) and with a configuration of six filters ( 755LP, 685LP, 670LP, 635LP, 600LP, 550LP and 505LP) on a blue laser (488 nm), three filters (595LP, 505LP and 450/50) on a violet laser (405 nm) and three filters (755LP, 685LP and 670/30) on a red laser (633 nm). FCS files were analysed using FlowJo (v10.7, Tree Star).

### T_FR_ counts and cell death time course

For the 5-day time course, cells were prepared and cultured as above with the exception of addition of 10 U ml^−1^ of IL-2 to culture. Cells were harvested and analysed each day for cell death and total counts. Cell counts were performed using CountBright absolute counting beads (Molecular Probes, C36950) according to the manufacturers’ instructions. Briefly, all cell fractions were resuspended in 400 μl of buffer and 50 μl of beads were added. At least 1,000 bead events were collected for each sample and the number of cells per μl was calculated based on the fixed bead concentration supplied by the manufacturer. In addition, a separate analysis of the same cell fractions was performed using the Annexin-V PE apoptosis detection kit (eBioscience, 88–8102) according to the manufacturers’ instructions. Each T-cell population was analysed based on early apoptosis (Annexin-V staining), late or advanced apoptosis (Annexin-V and PI) and necrosis (PI).

### ELISAs

Cell culture supernatants were measured for TGF-β-1 secretion (R&D Systems, DB100B), IL-10 (R&D Systems, DB1000B), IFNy (R&D Systems, DIF50) and IDO (Cloud Clone SEB547Hu) according to the manufacturers' instructions. Absorbance values were converted to protein levels using a protein standard dilution. Cell lysates as well as culture supernatants were evaluated by IDO ELISA.

### Intracellular cytokine staining assays

After 2 days of culture, tonsil cells were stimulated with 50 ng ml^−1^ of PMA (Sigma, P8139) and 1 μg ml^−1^ of ionomycin (Sigma, I3909) in the presence of protein transport inhibitor containing monensin (BD GolgiStop) for 5 h. Cryopreserved rhesus macaque cells were rested for 24 h (viability 40–80%) in R10 and then stimulated in the same manner for 5 h. Cells were then harvested, blocked and stained for surface markers as above, and then fixed and permeabilized using BD CytoFix/Cytoperm kit (554714) according to the manufacturer's instructions. Cells were then stained at 4 °C for 30 min with IL-4-PercpCy5.5-8D4–8 (BD, 561234), IL-10-eFluor450-JES3–9D7 (eBioscience, 48–7108), IL-21-AF647-3A3-N2.1 (BD, 560493) and TGF-β-1-PE(LAP)-27232 (R&D, FAB2463P). All antibodies were used at one test per 10^6^ cells. All cytokine analyses were normalized to a mock-spinoculated control that received monensin but was unstimulated.

### CD25-depletion cultures

CD25^+^ cells were depleted immediately after tonsil cell disaggregation using a FITC-positive selection kit (StemCell, 18558) and CD25-FITC-BC96 (BioLegend, 302604). Cells were spinoculated and cultured as described above. Mock- and HIV-spinoculated cultures from non-depleted cells were run in parallel with each experiment. After 2 days of culture, cells were analysed for T_FH_ and T_FR_ phenotypes by flow cytometry as described above. A subset of CD25^+^-depleted cells was analysed to confirm the absence of Foxp3^+^ cells.

### BrdU *ex vivo* proliferation assay

*Ex vivo* BrdU labelling was performed using a flow cytometry kit (eBioscience, 8817-6600) according to the manufacturers’ instructions. For BrdU labelling of primary cells in culture, a range of incubation times for BrdU uptake were tested and 24 h was found to be the optimal timepoint. Tonsil cells were spinoculated and cultured as above and labelled with 10 μM of BrdU for 24 h before analysis. After 2 days of culture, cells were collected, stained with surface antigens, fixed and permeabilized and treated with DNase I to allow BrdU antibody binding.

### Cell sorting and T_FR_/T_FH_ co-culture

Disaggregated tonsil cells were sorted using a MoFlo Astrios EQ. Cells were sorted into non-follicular (CD3^+^CD8-CXCR5^−^), T_FH_ (CD3^+^CD8-CXCR5^+^CD25^−^) and T_FR_ (CD3^+^CD8-CXCR5^+^CD25^hi^) populations. A subset of non-follicular and T_FH_ cells were cultured either without treatment or with 100 ng ml^−1^ of exogenous TGF-β to analyse whether T_FH_ convert to T_FR_. After sorting, T_FH_ and T_FR_ were spinoculated with X4 or R5 HIV GFP reporter viruses. T_FH_ were then seeded at a set number of 1 × 10^5^ cells in a 24-well plate and T_FR_ were added at ratios of 1:1, 1:10 and 1:50. After 2 days, cells were stimulated to measure IL-4 and IL-21 by ICS or left unstimulated to measure ICOS expression. In select experiments, T_FH_ and T_FR_ were co-cultured in the presence of neutralizing antibodies to IL-10 (1 μg ml^−1^; BioLegend, 501406) and TGF-β (1 μg ml^−1^, BioLegend, 521704).

### Proliferation assays

T_FH_ (CD3^+^CD8-CXCR5^+^CD25^−^) were sorted and stained with proliferation dye (Cell Proliferation Dye eFluor670, eBioscience, 65–0840) at a concentration of 0.5 μM. In a 96-well plate, pre-coated with 5 μg ml^−1^ anti-CD3 (Tonbo, 40-0037) in PBS at 37 °C for 2 h, 10^4^ T_FH_ per well were cultured for 4 days in 200 μl R10 containing 2 μg ml^−1^ anti-CD28 (Tonbo, 40–0289) and 10 U ml^−1^ IL-2 with an equal number of sorted T_FR_ (CD3^+^CD8-CXCR5^+^ CD25^+^CD127^−^) or alone. At day 4, cells were stained with viability dye (Ghost V450, Tonbo, 13–0863) and analysed by flow cytometry.

### Statistical analysis

Comparisons of infected and seronegative human LNs or rhesus macaque spleen or LNs were performed by non-parametric Mann–Whitney tests. Comparisons of tonsil cultures were performed by unpaired Mann–Whitney or Friedman nonparametric tests. In direct comparisons of paired data, a paired Wilcoxon ranked sums test was performed to compare the two group medians of interest. Significance is denoted in each figure by asterisks, as *P<0.05, **P<0.01 and ***P<0.001.

## Additional information

**How to cite this article:** Miles, B. *et al*. Follicular regulatory T cells impair follicular T helper cells in HIV and SIV infection. *Nat. Commun.* 6:8608 doi: 10.1038/ncomms9608 (2015).

## Supplementary Material

Supplementary Information Supplementary Figures 1-4
