# Supplementary material for: Follicular regulatory T cells impair follicular T helper cells in HIV and SIV infection
Source: Nat Commun. 2015 Oct 20;6:8608. doi: 10.1038/ncomms9608 (PMC4616158; doi:10.1038/ncomms9608)
Supplement: Supplementary Information — Supplementary Figures 1-4 [file ncomms9608-s1.pdf]

Supplementary Figure 1

a

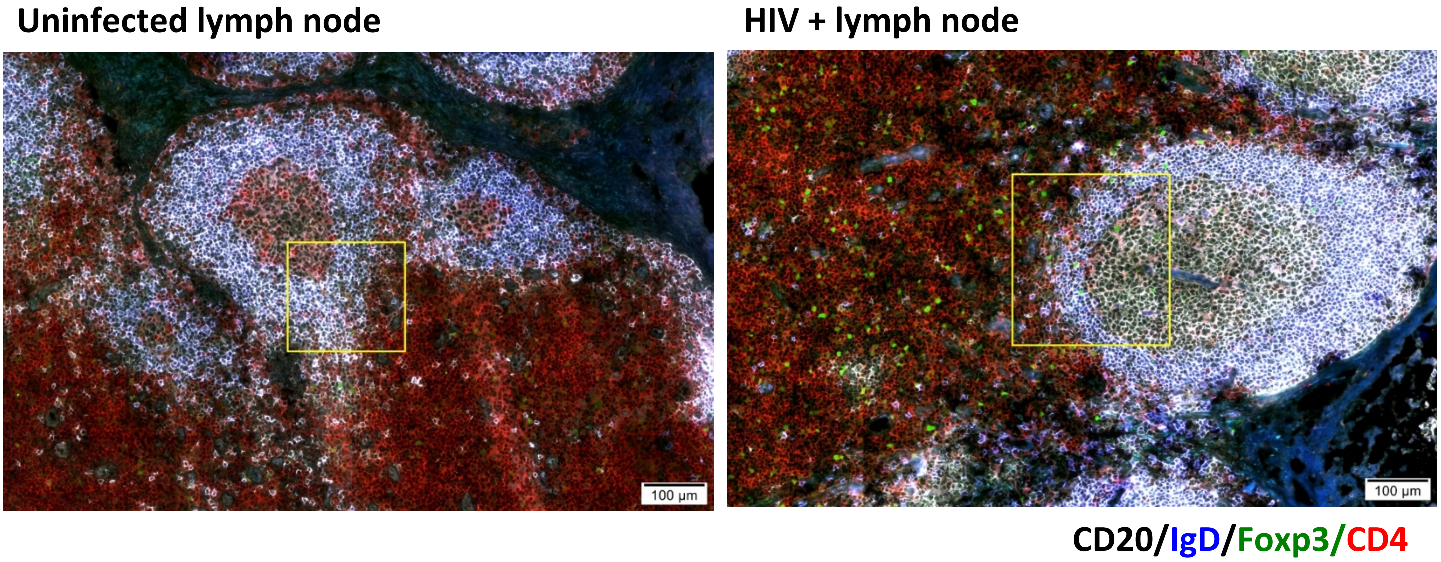

b

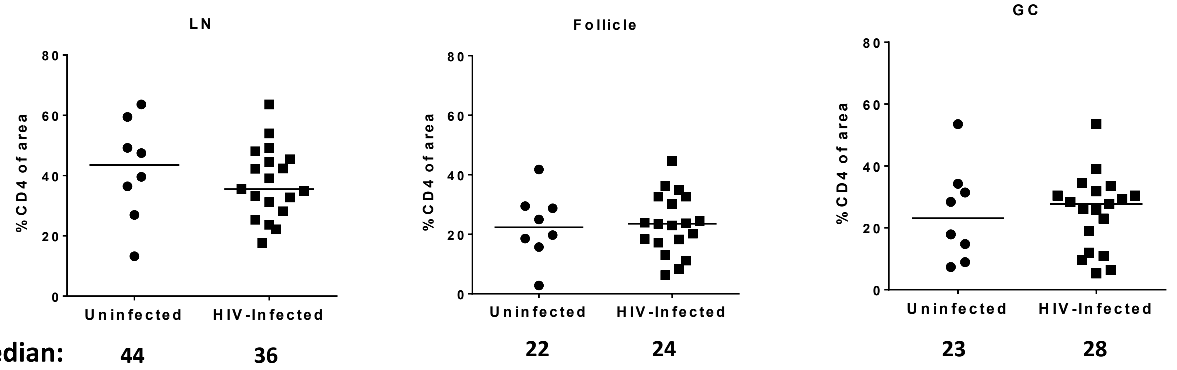

c

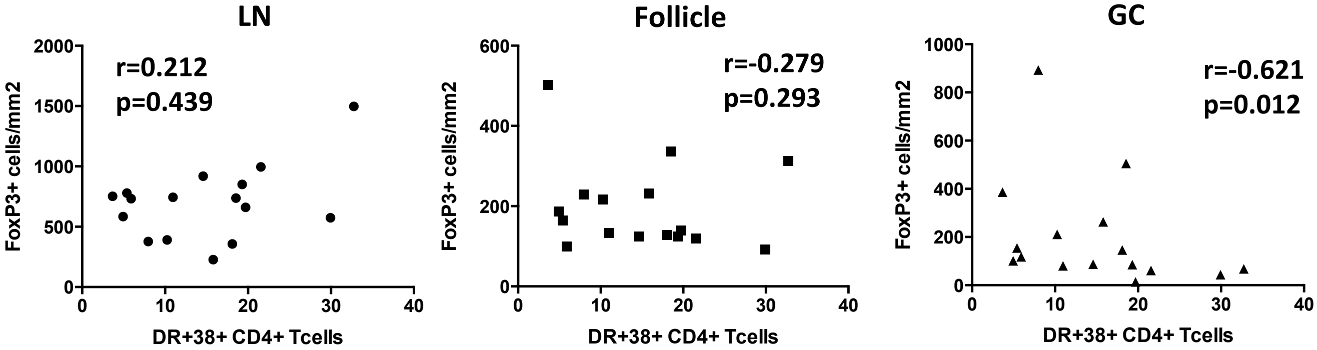

Supplementary Figure 1. Analyses of immunofluorescently stained human lymph nodes. (a) Representative immunofluorescent images of lymph nodes from an HIV uninfected and an HIV-infected individual. Lymph nodes were stained for CD20 (white), IgD (blue), Foxp3 (green), and CD4 (red). Yellow squares represent zoomed in images shown in Fig. 1a. Scale bars equal 100 µM. (b) The area of each region that stained as CD4+ was determined in uninfected (n=8) and HIV-infected (n=17) subjects using immunofluorescent staining shown in Fig. 1a. No significant changes between uninfected and HIV-infected individuals were detected. (c) The number of Foxp3+ cells per area were correlated to the number of CD38+HLA-DR+ cells for total, follicular, and germinal center lymph node regions. Statistical analyses were performed by Spearman correlation tests (c) to compare unpaired, nonparametric samples.

Supplementary Figure 2

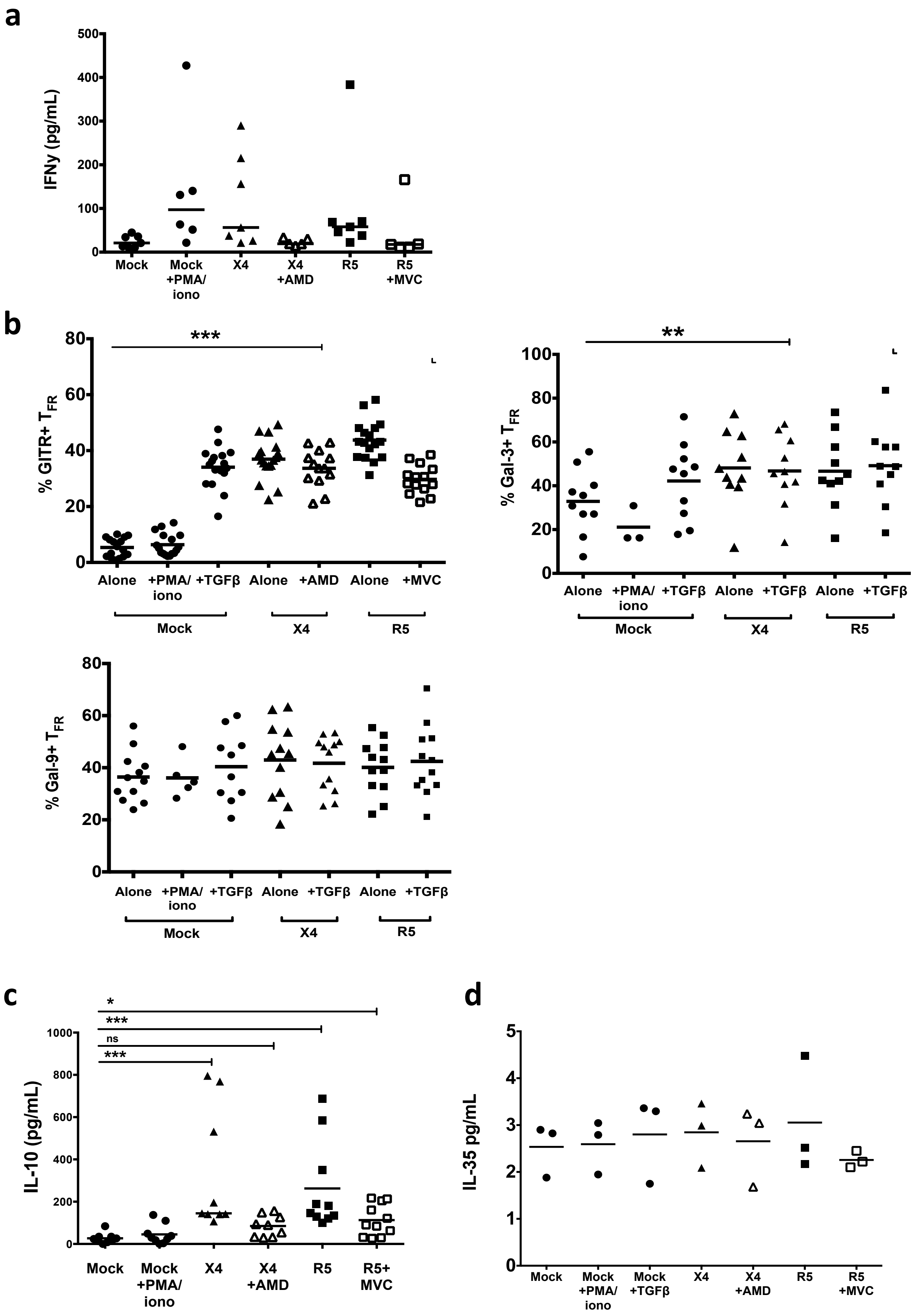

Supplementary Figure 2. Expression of regulatory molecules by T_{FR} in HIV infection *ex vivo*. Disaggregated tonsil cells were spinoculated and/or treated as indicated and analyzed for cytokine production (a,c, d) in culture or surface inhibitory molecules (b). (a) Culture supernatants were measured for IFNγ secretion by ELISA (n=7). (b) Percentages of T_{FR} surface expression of GITR (n=15), galectin-3 (n=10), and galectin-9 (n=12). (c) Culture supernatants were measured for IL-10 secretion by ELISA (n=9). (d) Culture supernatants were measured for IL-35 secretion by ELISA (n=3). The horizontal bars of each graph indicate the median value. Statistical analyses were performed by nonparametric Friedman tests and significance is denoted by asterisks where * = $p < 0.05$, ** = $p < 0.01$, and *** = $p < 0.001$.

Supplementary Figure 3

A

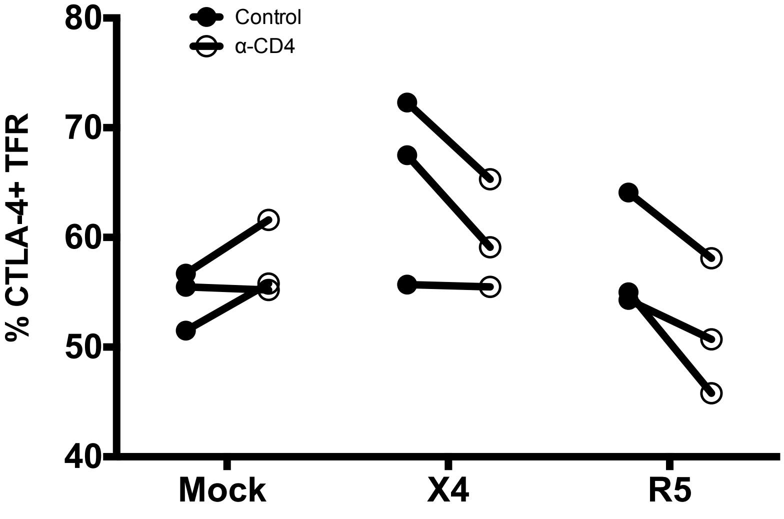

B

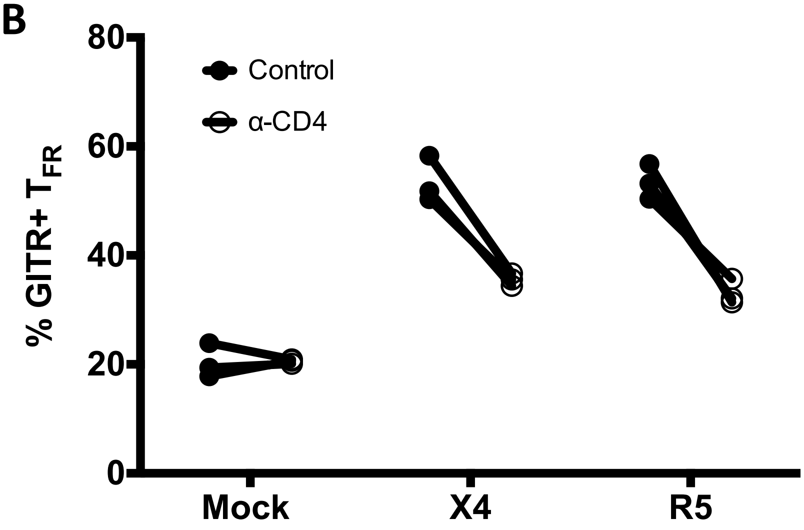

C

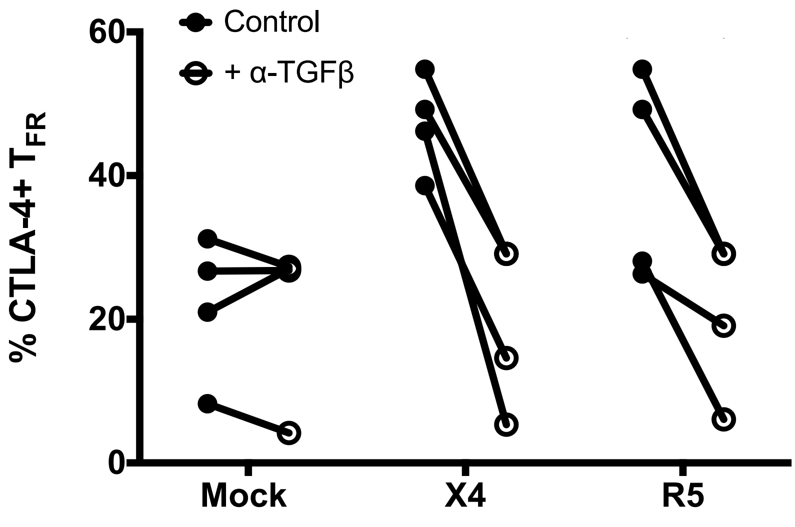

Supplemental Figure 3. CD4 and TGFβ signaling promote enhanced T_{FR} regulatory phenotype. Disaggregated tonsil cells were pretreated with a CD4-neutralizing antibody (10 ng/mL) prior to spinoculation to prevent X4- and R5-HIV infection and inhibitory receptors were analyzed after day 2 of culture. (a) Percent of total (surface and intracellular) CTLA-4 expression in T_{FR} with and without CD4 blockade (n=3). (b) Percent of GITR expression in T_{FR} with and without CD4 blockade (n=3). (c) Tonsil cells were treated with a TGFβ-neutralizing antibody (2 µg/mL) for the duration of culture. Percent of total CTLA-4 expression in T_{FR} with or without TGFβ blockade is shown (n=4).

Supplementary Figure 4

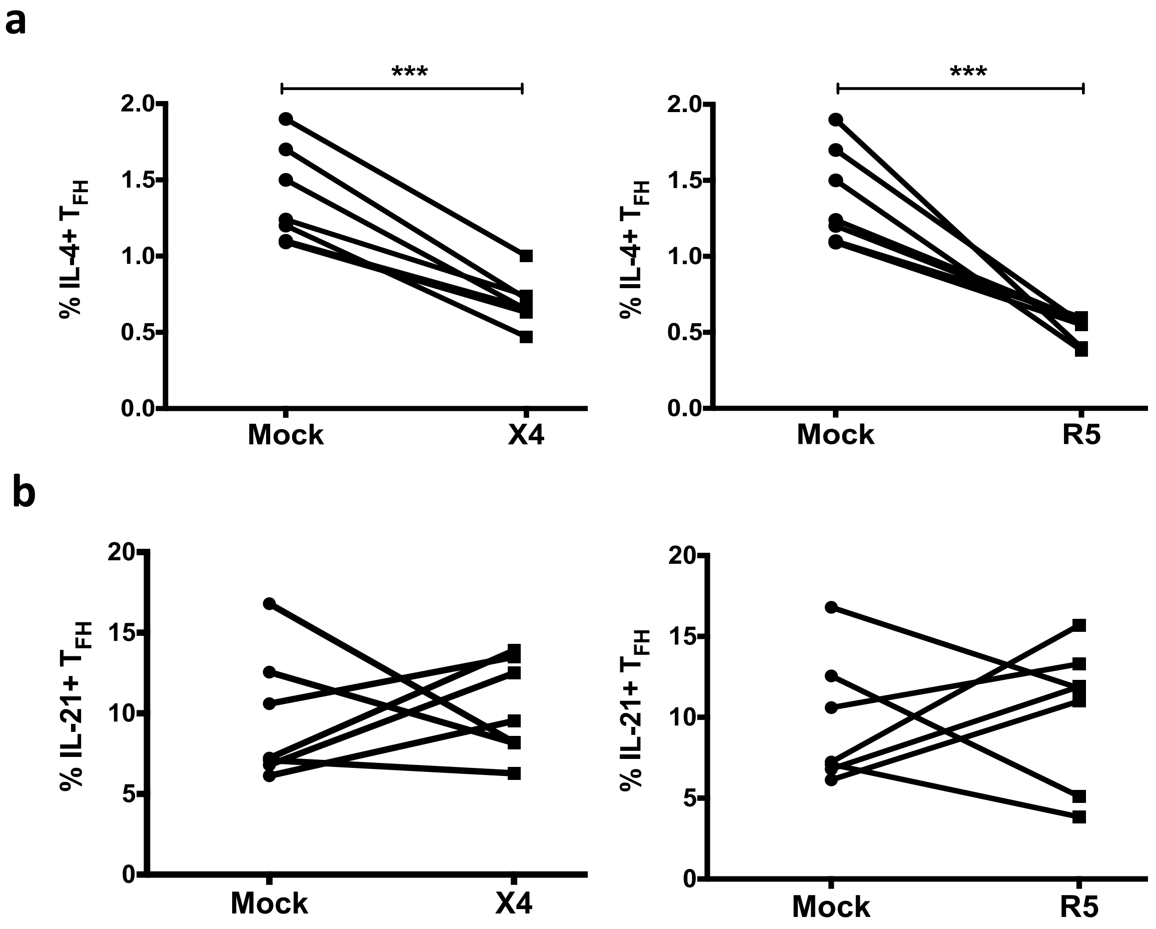

Supplemental Figure 4. IL-4 and IL-21 production by T_{FH} in whole tonsil cultures. (a) Tonsil cultures were mock-, X4-, or R5-spinoculated and cultured for 2 days. IL-4 production by T_{FH} was measured by intracellular cytokine production (n=7). (b) IL-21 was measured by intracellular cytokine production in the same cultures as (a) (n=7).
